# Optimization of extraction conditions for polyphenols from the stem bark of *Funtumia elastica* (Funtum) utilizing response surface methodology

**DOI:** 10.12688/aasopenres.13284.1

**Published:** 2021-09-10

**Authors:** Theophilus Fadjare Frempong, Nathaniel Owusu Boadi, Mercy Badu

**Affiliations:** 1 Department of Chemistry, Kwame Nkrumah University of Science and Technology, Kumasi, Ashanti Region, UPO PMB, Ghana

**Keywords:** polyphenols, maceration, Funtumia elastica, extraction factors, single factor

## Abstract

**Background:** The recovery of phenolic compounds is seen as an arduous task because phenolic compounds are available as free aglycones, as sugar or ester conjugates, or as polymers with several monomeric components. Furthermore, phenolic compounds do not disperse evenly and may be connected to cell walls, carbohydrates, or proteins. This study looks at the optimization of factors that affect the efficiency for the extraction of phenolic compounds from the stem-bark of *Funtumia elastica*.

**Methods: **Five independent variables (solvent concentration, time, the temperature, solid-liquid ratio, and pH) of the extraction process were selected. Single factor analysis as well as the response surface method was used to evaluate the impact of the selected factors on the total phenolic content. The effect of the extraction factors on the phenolic content was statistically significant (p <0.05). For the response surface method, a five/factor, five/level central composite design used, and a fitted second-order polynomial regression model equation was used to show how the extraction parameters affected the total phenolic recovery.

**Results: **The predicted value (R² of 0.5917) agreed with the adjusted value (R² of 0.7707). The residuals for response predictions were less than 5%. The optimal factors for the extraction were ethanol concentration of 75.99% v/v, extraction time of 193.86 minutes, temperature of 63.66°C, pH of 5.62, and solid-liquid ratio of 1:21.12 g/mL. Actual overall content of the phenolic compounds was validated at 82.83 ± 3.335 mg gallic acid equivalent (GAE) /g weight of extract, which agreed with the predicted response of 89.467 mg GAE/g of the dried extract under the optimal factors.

**Conclusions: **The rich phenolic content of stem-bark of *Funtumia elastica* points to its potential as a functional medicinal product to alleviate diseases caused by oxidative stress such as asthma, breathing disorders, inflammation, and cardiovascular diseases.

## Introduction

Phenolic compounds are naturally produced in plants for UV radiation protection, attracting pollinators and preventing microbial infections, etc. ^1^. These compounds act as natural antioxidants and are directly involved in lipid peroxidation and anti-carcinogenesis. The existence of diverse polyphenols acting as antioxidants facilitates the entrapment of free radicals spawned from various metabolic processes, thereby inhibiting the initiation of any cancerous cells ^2^. They also exhibit anti-inflammatory, antimicrobial, cardio-protective, and anti-aging activities ^3, 4^. Several reports have indicated a strong positive correlation between the total phenolic content (TPC) and the antioxidant activity of dietary and medicinal plants ^5, 6^.

Phenolic compounds from *Funtumia elastica* could be obtained using various conventional extraction methods—solid-liquid extraction by maceration, decoction, stirring, shaking ^7– 9^, using ethanol and water as their main solvents ^10, 11^. Other modern methods include microwave-assisted, ultrasonic ^12^, soxhlet, heat reflux ^11^, and ultrahigh-pressure ^13^ extractions. Despite the advantages of these modern extraction techniques, they are expensive to operate and ultrasound energy greater than 20kHz during ultrasound-assisted extraction may damage active phytochemicals ^14^. Moreover, thermal degradation and oxidation of compounds ^15^ are found in microwave-assisted extraction on account of the additional cycles of extraction and the fact that it is limited to small phenolic molecules ^14^.

Maceration is an easy and inexpensive extraction technique that utilizes simple equipment ^16^. Skilled operation is not needed compared to other modern techniques, and it ensures energy economy. This method is suitable and ideal for less soluble substances that require a prolonged interaction with the extraction solvent. Polyphenols are usually extracted using ethanol, methanol, or ethyl acetate as solvents ^17^. Ethanol with water is also widely used for extracting phenolics ^18^ and are commonly used by indigenous societies as their main solvents. During the maceration process, the efficiency of the extraction is attributed to numerous independent variables, such as the type of solvent, solvent concentration, temperature, pH, time of extraction, and the solid-liquid ratio ^19^. There is an interaction between these variables, which affects the efficiency of the extraction ^20^.

According to the one-factor-per-time (OFAT) method, all variables except one are kept constant at any point in time during the process ^21^. However, this technique is often met with numerous obstacles including: requiring a huge number of experimental runs; proving to be inefficient and unreliable in giving optimal factors; and the inability to study the interactions between the factors or factors affecting the process or product ^22^.

It is therefore imperative to assess the interaction between the independent variables. One of the appropriate techniques is to use the response surface method (RSM) as a tool to optimize the factors that affect extraction efficacy as well as to obtain maximum recovery of the compounds of interest ^23– 25^. The central-composite-design (CCD) of the response-surface-method is known to be useful for the optimization of extraction parameters of bioactive compounds ^26^.

The purpose of the study was to evaluate the effects of the investigated factors (ethanol concentration, extraction time, extraction temperature, solvent pH, and solid-liquid ratio) on the TPC of the hydroethanolic extract of stem bark of *Funtumia elastica* (Preuss) Stapf, a proven medicinal plant, and to optimize the extraction factors of the polyphenols further using CCD.

## Methods

### Plant materials used

The stem barks of *F. elastic*a from the tropical rain forest in the Asante Akim Municipality were collected by scraping them from intact mature trees. They were authenticated by a botanist at the Department of Herbal Medicine, KNUST. The samples were cleaned, chopped into pieces, and dried on plastic sheets under shade. The dried samples were milled using a pulverizer mill and kept in zip-locked bags and stored in the fridge at 4 ^o^C.

### Reagents

All chemicals and reagents used were of analytical grade. Folin-Ciocalteu, gallic acid, ethanol, and sodium carbonate were all obtained from Sigma-Aldrich.

### Maceration extraction

From the dry powdered sample, 10.00 g was extracted with a hydroethanolic solution in 250 to 500 mL screw-capped Erlenmeyer conical flasks. The extraction was conducted using a temperature-controlled water bath (Grant JBN12, Grant Instruments Ltd, Cambridge, UK). The extraction conditions were set according to the experimental design. All the extractions were carried out in replicates.

### Determination of TPC

The TPC of the plant extracts was measured spectrophotometrically using the Folin-Ciocalteu method previously reported ^27^. Briefly, an amount of 0.5 mL of each solution of the different extracts (500–1000 µg/mL) was measured into test tubes, and 2.5 mL of Folin-Ciocalteu reagent added to each. 2 mL of aqueous sodium carbonate solution (75 mg/mL) was added to each and kept on a water bath at 50°C for 10 mins. The absorbance was read at 760 nm using a microplate reader (Synergy H1 ^TM^ Microplate Spectrophotometer, Thermo Fisher Scientific, Osaka, Japan). The gallic acid solutions were taken through the same procedure and used to plot a calibration curve of absorbance against log concentration (μg/mL). The total phenolic content was expressed as mg gallic acid equivalent (GAE) per gram of extract.

### Experimental design

The experimental procedure of the study was group into two parts; a single-factor analysis and optimization using RSM.

***Single-factor analysis.*** To determine a suitable range of factors, single-factor analysis was conducted using solvent concentration (20, 40, 60, 80 and 96% v/v), time for extraction (60, 120, 180, 240, and 300 minutes), extraction temperature (25, 35, 45, 55, and 65°C), pH (2.5, 5.5, 7, 9, and 12), and solid-liquid ratio (1:10, 1:20, 1:30, 1:40, and 1:50 g/mL). One independent variable was varied according to the set range whilst keeping the others constant. Based on the results obtained, all the five independent variables had significant influence on the extraction efficiency and were subsequently selected for the RSM design.

***RSM.*** This study deployed a five-factor, five-level CCD model (-2.378, -1, 0, +1, +2.378) to examine the optimum conditions for the dependent variable or response; total polyphenolic content maximization. The design comprised 50 randomized runs with 32 factorial points, 10 axial points, and eight replicates as the center points. The center points were repeated eight times to ascertain the statistical parameters of the proposed models. The five independent variables with their related actual and coded values are shown in Table 1.

**Table 1.** Five-factor-five-level independent variables of the extraction process.

Independent variable	Units	Symbol	Coded values				
			-2.37841	-1	0	1	2.37841
Ethanol concentration	%(v/v)	X _1_	46.22	60.00	70.00	80.00	93.78
Time	minutes	X _2_	138.65	180.00	210.00	240.00	281.35
Temperature	°C	X _3_	31.22	45.00	55.00	65.00	78.78
pH		X _4_	3.10	5.50	7.25	9.00	11.41
Solid-liquid ratio	g/mL	X _5_	13.11	20.00	25.00	30.00	36.89

The range of each level were ethanol concentration (46.22 - 93.78% (v/v), temperature (45 - 65°C), time (138.65 - 281.35 minutes), pH (3.10 - 11.41) and solid-liquid ratio (1:13.11 - 1:36.89 w/v). The data from the experiment were analyzed by multiple regression fitted to the following second-order polynomial equation:

$$Y=\beta_{0}+\sum \beta_{i}X_{i}+\sum \beta_{ii}X_{i}^{2}+\sum \beta_{ij}X_{i}X_{j}$$

where X _i _and X _j_ represent the independent variables. β _o_, β _i_, β _ii_ and β _ij_ (i≠j) represent the coefficient of regression for the intercept (constant), linear, quadratic, and interaction terms, respectively. Design-Expert Software 11.0.0 (Stat-Ease, Minneapolis, MN, USA) was used to build the model, calculate the predicted values, and plot the three-dimensional graphs. Other tools such as Scilab, OpenDino and JMP statistical software could also be used. 

### Validation of the optimized model

For validation of the optimized extraction parameters, six different experimental setups were employed for a trial extraction process. The average results (value) obtained from the trial extraction was compared with the predicted values.

### Statistical analysis

The significance of the model and equation terms was investigated by determining the analysis of variance (ANOVA) with a 95% confidence interval. The statistical parameters; the sum of squares (SS), degrees of freedom (df), mean squares (MS), p-values, and F-values were employed. The model terms were deemed statistically significant if p <0.05. The quality of the regression model was shown using the coefficient of determination (R ^2^). To express the quality of the regression model, the R ^2^, adjusted correlation coefficient (Adj-R ^2^), predicted correlation coefficient (Pred-R ^2^), and the significance of the model were tested using F-test.

In this study, all the experiments were performed in triplicate, and the results were expressed as mean value ± standard deviation (SD). The statistical analysis was carried out using Excel 2019 (Microsoft, Redmond, WA, USA) and Design-Expert version 11.0.0. Other tools such as Scilab, OpenDino and JMP statistical software could also be used. Multiple comparisons were carried out by ANOVA plus the posthoc Tukey test. p <0.05 was defined as statistical significance.

## Results and discussion

### Effect of single factor analysis of the TPC

**Figure 1.**
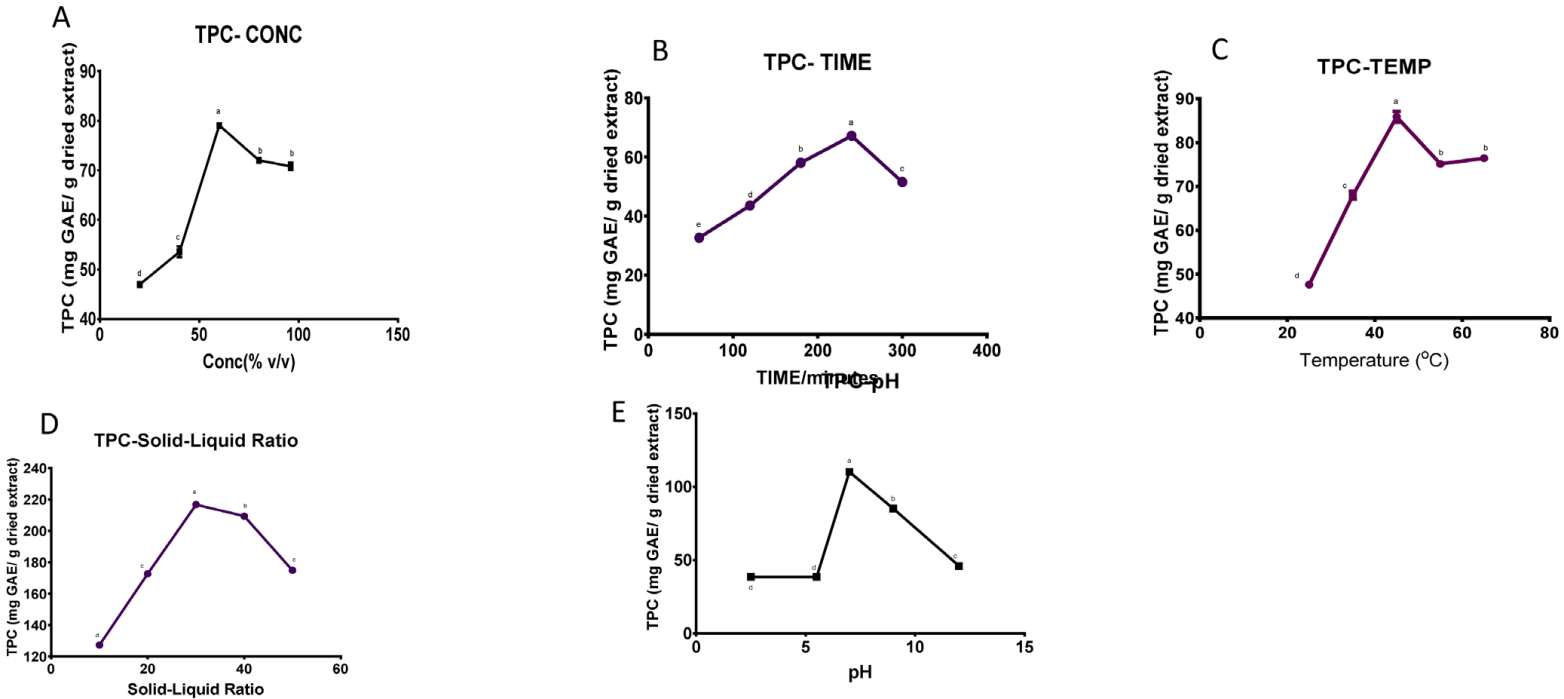
The effect of ( **a**) solvent concentration ( **b**) extraction temperature ( **c**) time ( **d**) solid-liquid ratio and ( **e**) pH on the total phenolic content of *F. elastica* (n= 3). Values are presented as means ± standard deviation of six measurements. Values marked by different lower-case letters ( **a**– **e**) are significantly different ( *p* <0.05)

The impact of ethanol concentration, extraction time, temperature, solid-liquid ratio, and pH on the TPC from *F. elastica* stem bark is shown in Figure 1a–e.

***Effect of the ethanol concentration.*** The effect of the concentration of ethanol, ranging from 20% to 96% v/v, on the TPC is illustrated in Figure 1a. The extraction was conducted at a solid-liquid ratio of 1:20, temperature of 35°C, time of 180 mins, and extraction pH of 7.00. The recovery increased with an increased ethanol concentration and reached a maximum at 60% ethanol (79.062 ± 0.691 mg GAE/g of dried extract, p <0.05) until it showed a declining tendency from 60 to 96% v/v ^28^. As a result, a range of 60-80% v/v was chosen for the optimization experiment.

An ethanol:water mixture was used as the solvent for this study. Compared with other solvents, ethanol is a relatively low-cost solvent, is readily available, and is widely recognized as a green solvent. For extraction to be effective, the solvent polarity should correspond with the targeted phytoconstituents ^29^ **.** The polarity results in the solvent solubilizing the target analytes while leaving the sample intact. When solvents of different polarities are mixed, they tend to extract a wide spectrum of compounds ^30^. Chew *et al.* (2011) performed a single factor analysis on optimization of TPC recovery from *Orthosiphon stamineus* by investigating the impacts of three independent variables. Their results showed a significant effect (p <0.05) on TPC. The TPC level rose as ethanol concentration was increased to 40% v/v. The TPC recovery level decreased significantly beyond 40% v/v until reaching 100% v/v (p <0.05) ^31^. Elboughdiri (2018) investigated the influence of ethanol concentration of TPC yield in olive leaves using ethanol concentrations of 50%, 60%, 70%, and 80% (v/v). However, they reported no significant difference between the recovered TPC levels of the four ethanol concentrations ^32^, which was consistent with our results.

***Effect of extraction time.*** The effects of the different extraction times on TPC of the extract was investigated in the range of 60 to 300 minutes. The other independent variables that were kept constant were the maximum 60% ethanol concentration from the first analysis, 35°C temperature, pH of 7, and a solid-liquid ratio of 1:20 g/mL. The impact of different extraction times on the TPC was seen in Figure 1b, which shows an increase in the TPC as the extraction time increased to 240 minutes. The maximum TPC recovery was observed at 240 minutes as 67.219 ± 0.642 mg GAE/g of dried extract for *F. elastica.* There was, however, a decline in TPC from 240 to 300 minutes. Thus, a range of 180 to 240 minutes was chosen for the optimization process.

Several research articles have reported a decrease in total phenolics content as the extraction time was prolonged ^33^. Jia *et al.* (2019) reports the significant effect of enzymolysis time (p <0.05) on anthocyanin extraction from cherry wine lees. The yield of anthocyanins reached a maximum at 50 minutes and declined as the time increased ^34^. Le *et al.* (2019) also presented the impact of maceration extraction time on TPC from *Glycine max L*. TPC gradually increased from 5.4 to 12.8mg GAE/g of dried extract as the time increased from 15 to 60 minutes. However, from 60 to 150 minutes, the TPC remained unchanged at around 12.7 mg GAE/g of dried extract. ^35^. Fick’s second law of diffusion could explain this phenomenon by stating that an equilibrium was established between the solute and the extraction solvent ^36^. As a result, when the time was prolonged, it did not significantly impact the TPC extraction ^35^. One of the essential independent variables in solid-liquid extraction is time due to its impact on the analyte’s solubility and mass transfer which is associated with the compound’s structure and molecular weight **.** Prolonged extraction time may lead to oxidation, epimerization, and degradation of the metabolites of interest ^37^. This was observed in our study, as shown in Figure 1b.

***Effect of extraction temperature.*** The influence of the extraction temperature on TPC recovery is shown in Figure 1c. A significant (p <0.05) impact on the phenolic content of the extract was observed when varying the temperature from 25°C, 35°C, 45°C, 55°C, 65°C while keeping the other independent factors constant, with the solid:liquid ratio at 1:20 g/mL, ethanol concentration at 60%, extraction time at 240 minutes based on the previous study and pH at 7.00. The highest peak was seen at 45°C for *F. elastica* (85.84± 2.16 mg GAE/g of dried extract), and the TPC started to decline when the temperature was further increased. Consequently, a temperature range of 45-65°C was chosen for the optimization study.

An increase in temperature significantly increases the mass diffusivity ^38^, which increases the rate at which the solute diffuses into the solvent.

In addition, other reports ^39^ emphasize that high extraction temperatures increase the mass transport of phenolic compounds and even reduce solvent viscosity and surface tension, thereby promoting phenolic compound extraction. The heating may, in turn, soften plant tissues, which weakens the phenol-polysaccharide and other phenol interactions and foster the migration or leaching of phytochemicals like flavonols into the solvent ^40^. The solubility depends on the entropy of fusion, melting point of the solute, and the activity coefficient of the mixture. Low melting point and high temperature lead to increased solubility ^41^. However, high temperatures can affect the stability of some phenolic compounds, including anthocyanins ^42^. According to Schwartzberg and Chao (1982) ^43^, temperatures from 55°C to 75°C can cause denaturing of membranes and affect the extraction process. Therefore, it is not advisable to increase the temperature indefinitely. To optimize extraction, a compromise temperature should be determined ^44^. Unsurprisingly, in our case, we observed a decline in phenolic content from 55°C and 65°C in *F. elastica*. Similar reports have shown maximum anthocyanin content peaking at 35°C, and a sharp decrease in anthocyanins at temperatures higher than 45°C ^45^. Other researchers stated a decline in antioxidants when the temperature exceeded 40°C. The yield of antioxidants may have dropped due to the degradation of some heat-labile compounds at higher temperatures ^16, 46^. Chew *et al.* (2011) reported the yield of phenolic compounds as increasing with increasing extraction temperature ^31^, which was not consistent with our findings. Increased temperatures promote extraction by improving phenolic solubility and increasing the diffusion coefficient, which increases the extraction rate and reduces time ^45^.

***Effect of solid-liquid ratio.*** In Figure 1d, five different solid-liquid ratios (1:10, 1:20, 1:30, 1:40, and 1:50) were examined whilst keeping the other factors constant as follows; 60% ethanol concentration, 45°C extraction temperature, 240 minutes duration and pH of 7.00. The results show that TPC is significantly (p <0.05) impacted by the solid-liquid ratio. A trend was observed with the TPC peaking at 1:30 (216.786 ± 0.200 mg GAE/g of dried extract). The TPC yield displayed a decreasing tendency from 1:30 to 1:50 g/mL. Thus, a range from 1:20 to 30 g/mL was selected for the optimization process.

Theoretically, the solid-liquid ratio significantly affects the extraction kinetics of the bioactive compounds due to its effect on the concentration gradient between the solute and the solvent at the surface of the raw material ^47^. Many authors have reported a strong positive correlation between the TPC and solid-liquid ratio. A study conducted varied the solid-liquid ratio from 1:04 to 1:10 and observed a gradual increase in TPC from 1:04 to 1:06 but achieved equilibrium beyond 1:06 to 1:10 ^35^. This could be explained by an increase in concentration gradient as more solvent is added. However, the addition of ethanol will stop promoting TPC yield when the content of polyphenols in the material is depleted ^35^.

Wang *et al.* (2020) also demonstrated the influence of solid-liquid ratio on the yield of the polysaccharides from *Bletilla ochracea* Schltr. The yield was improved when the solid-liquid ratio (1:10–1:30 g/mL) was increased and the optimum value for 1:30 g/mL was reached when temperature and time were set to 80°C min. and 90 minutes. The yield, however, showed a decreasing tendency between 1:30–1:50 g/mL ^48^.

Another author determined the factors influencing the extraction of polyphenols from olive leaves. Three solid-liquid ratios (1:20 g/mL, 1:25 g/mL and 1:30 g/mL) were considered. The results of the study showed that the solid-liquid ratio affected the overall phenol levels significantly. The higher solvent-to-solid ratio (1:30 g/mL) achieved the highest TPC (24.5 mg caffeic acid/g of dry matter) ^32^.

In general, a higher solid-liquid ratio could cause a greater concentration difference, which will in turn increase the diffusion of components into solvents and accelerate mass transfer ^49^.

***Effect of extraction pH.*** The influence of pH, which ranged from 2.5 to 12, was evaluated using a 60% ethanol solution, a solid-liquid ratio of 1:20 and temperature of 45 ^o^C, and time for extraction was 240 minutes, as shown in Figure 1e. The TPC of *F. elastica* peaked at a pH of 7.00 (110.119 ± 0.200 mg GAE/g of dried extract, p <0.05). There was a decline in TPC as the pH increased from 9.00 to 12.00. The decrease could be due to the degradation of the phenolics or lower extraction under basic conditions. The TPC of *Ocimum sanctum* methanolic leaf extract was investigated under different pH conditions, and the highest TPC was observed at a pH of 7.20 ^50^. Our results were consistent with the maximum TPC of 7.20 reported in literature.

The antioxidant capacity and radical scavenging ability of many phenolic compounds are based on the number and positions of the hydroxyl groups and the methoxy substituents in molecules ^51^. Changes in pH, like temperature conditions, can modify the phenolic compounds' chemical structure and thus the antioxidant activity of the compounds.

The antioxidant properties are pH-dependent, as changes in pKa values are consistent with changes in hydroxyl ionization or other functional groups of the phenolic compounds ^20^.

### Modeling of the total phenolic recovery using RSM-CCD

A RSM-CCD with five independent variables and five levels was applied to evaluate the impact of vital extraction variables, including the concentration of ethanol (X _1_), extraction time (X _2_), extraction temperature (X _3_), pH (X _4_) and solid-liquid ratio (X _5_), on TPC recovery of *F. elastica*.

The experimental design, experimental values, and predicted values of the 50 runs are shown in Table 2 for both plants. The TPC value ranged from 2.14821 to 124.391 mg GAE/g of dried extract for *F. elastica*.

**Table 2.** The experimental design, actual value, and predicted value of response surface method.

Std. order	Run	X _1_ (Ethanol conc., % v/v)	X _2_ (Extraction time, mins)	X _3_ (Temperature ^o^C)	X _4_ (pH)	X _5_ (Solid-liquid ratio, g/mL)	TPC, mg GAE/g of dried extract	
							Actual value	Predicted value
42	1	70.00	210.00	55.00	7.25	36.89	38.62	50.32
15	2	60.00	240.00	65.00	9.00	20.00	106.72	111.20
24	3	80.00	240.00	65.00	5.50	30.00	100.44	107.51
11	4	60.00	240.00	45.00	9.00	20.00	79.99	75.37
18	5	80.00	180.00	45.00	5.50	30.00	45.67	39.66
49	6	70.00	210.00	55.00	7.25	25.00	98.58	88.05
10	7	80.00	180.00	45.00	9.00	20.00	31.74	33.93
35	8	70.00	138.65	55.00	7.25	25.00	74.37	61.52
9	9	60.00	180.00	45.00	9.00	20.00	74.81	70.92
43	10	70.00	210.00	55.00	7.25	25.00	78.72	88.05
30	11	80.00	180.00	65.00	9.00	30.00	53.54	48.59
50	12	70.00	210.00	55.00	7.25	25.00	101.32	88.05
29	13	60.00	180.00	65.00	9.00	30.00	66.62	76.25
44	14	70.00	210.00	55.00	7.25	25.00	99.62	88.05
22	15	80.00	180.00	65.00	5.50	30.00	66.79	76.84
45	16	70.00	210.00	55.00	7.25	25.00	68.87	88.05
13	17	60.00	180.00	65.00	9.00	20.00	97.48	90.46
23	18	60.00	240.00	65.00	5.50	30.00	93.85	86.07
7	19	60.00	240.00	65.00	5.50	20.00	94.27	97.02
41	20	70.00	210.00	55.00	7.25	13.11	79.89	64.54
27	21	60.00	240.00	45.00	9.00	30.00	64.22	72.08
4	22	80.00	240.00	45.00	5.50	20.00	65.87	51.77
6	23	80.00	180.00	65.00	5.50	20.00	105.19	85.51
2	24	80.00	180.00	45.00	5.50	20.00	22.19	44.73
31	25	60.00	240.00	65.00	9.00	30.00	113.77	104.32
33	26	46.22	210.00	55.00	7.25	25.00	91.75	88.63
8	27	80.00	240.00	65.00	5.50	20.00	112.92	108.86
14	28	80.00	180.00	65.00	9.00	20.00	37.05	53.19
40	29	70.00	210.00	55.00	11.41	25.00	15.62	11.49
20	30	80.00	240.00	45.00	5.50	30.00	48.91	54.02
1	31	60.00	180.00	45.00	5.50	20.00	16.30	29.76
21	32	60.00	180.00	65.00	5.50	30.00	46.79	52.55
25	33	60.00	180.00	45.00	9.00	30.00	47.88	60.31
12	34	80.00	240.00	45.00	9.00	20.00	39.15	35.53
3	35	60.00	240.00	45.00	5.50	20.00	32.65	39.66
48	36	70.00	210.00	55.00	7.25	25.00	68.08	88.05
32	37	80.00	240.00	65.00	9.00	30	92.78	73.81
37	38	70.00	210.00	31.22	7.25	25.00	33.70	33.80
46	39	70.00	210.00	55.00	7.25	25.00	103.66	88.05
39	40	70.00	210.00	55.00	3.09	25.00	2.15	2.62
5	41	60.00	180.00	65.00	5.50	20.00	60.21	70.82
34	42	93.78	210.00	55.00	7.25	25.00	70.69	70.14
26	43	80.00	180.00	45.00	9.00	30.00	36.48	32.93
47	44	70.00	210.00	55.00	7.25	25.00	87.99	88.05
16	45	80.00	240.00	65.00	9.00	20.00	50.76	71.09
28	46	80.00	240.00	45.00	9.00	30.00	43.05	41.85
19	47	60.00	240.00	45.00	5.5	30.00	38.96	32.30
36	48	70.00	281.35	55.00	7.25	25.00	94.09	103.28
38	49	70.00	210.00	78.78	7.25	25.00	124.39	120.64
17	50	60.00	180.00	45.00	5.50	30.00	36.28	15.08

Std. order: Standard order; TPC, total phenolic content; GAE, gallic acid equivalent.

***Fitting the model.*** The data were investigated by multiple regression fitting and second-order quadratic polynomial regression model equations based on the coded values. These were automatically generated by the Design-Expert software. The equations communicate the relationship between the value of the response and the five independent factors, as shown in Table 3. The equation in terms of coded factors can be used to make predictions about the response for given levels of each factor. By default, the high levels of the factors are coded as +1, and the low levels are coded as -1. The coded equation is useful for identifying the relative impact of the factors by comparing the factor coefficients.

**Table 3.** Response variables and their fitted model equations.

Symbol	Response variable	Quadratic equation
Y _TPC_ ( *F. elastica*)	Total phenolic content (mg GAE / g of dried extract	TPC = 88.05 -3.89X _1_ + 8.78 X _2_ + 18.26 X _3_ + 1.86 X _4_ -2.99 X _5_ -0.7135 X _1_X _2_ – 0.0690 X _1_X _3_ – 12.99 X _1_X _4_ + 2.40 X _1_X _5_ + 4.07 X _2_X _3_ -1.36 X _2_X _4_ + 1.83 X _2_X _5_ – 5.38 X _3_X _4_ - 0.8992 X _3_X _5_ +1.02 X _3_X _4_ -1.53 X _1_^2^ -0.9999 X _2_^2^ -1.92 X _3_^2^ – 14.32 X _4_^2^ -5.41 X _5_^2^

TPC, total phenolic content; GAE, gallic acid equivalent.

***ANOVA for the quadratic model.*** ANOVA was utilized to establish the strongest correlation between each response to indicate that the contribution of the quadratic model was significant and fit the data from the experiment. The data for the ANOVA are shown in Table 4. The ANOVA determined the significance of the model, constant terms, linear terms, interaction terms, and square terms. Hence, the quadratic model was employed to predict the TPC based on the data from the 50 runs.

**Table 4.** ANOVA for quadratic model of *F. elastica*.

Source	Sum of squares	df	Mean square	F-value	p-value	
**Model**	38465.18	20	1923.26	9.24	< 0.0001	significant
X _1_-Ethanol concentration	654.28	1	654.28	3.14	0.0868	
X _2_-Extraction time	3337.70	1	3337.70	16.03	0.0004	
X _3_-Temperature	14435.00	1	14435.00	69.33	< 0.0001	
X _4_-pH	150.64	1	150.64	0.7235	0.4020	
X _5_-Solid-iquid ratio	386.79	1	386.79	1.86	0.1834	
X _1_X _2_	16.29	1	16.29	0.0782	0.7817	
X _1_X _3_	0.1523	1	0.1523	0.0007	0.9786	
X _1_X _4_	5398.25	1	5398.25	25.93	< 0.0001	
X _1_X _5_	184.58	1	184.58	0.8865	0.3542	
X _2_X _3_	531.33	1	531.33	2.55	0.1210	
X _2_X _4_	59.39	1	59.39	0.2852	0.5974	
X _2_X _5_	107.24	1	107.24	0.5151	0.4787	
X _3_X _4_	926.85	1	926.85	4.45	0.0436	
X _3_X _5_	25.88	1	25.88	0.1243	0.7270	
X _4_X _5_	33.12	1	33.12	0.1590	0.6930	
X _1_²	130.41	1	130.41	0.6263	0.4351	
X _2_²	55.55	1	55.55	0.2668	0.6094	
X _3_²	203.88	1	203.88	0.9792	0.3306	
X _4_²	11392.56	1	11392.56	54.72	< 0.0001	
X _5_²	1628.66	1	1628.66	7.82	0.0091	
**Residual**	6038.22	29	208.21			
Lack of fit	4520.59	22	205.48	0.9478	0.5765	not significant
Pure error	1517.62	7	216.80			
**Cor Total**	44503.40	49				

**Response:** total phenolic content of *F.* elastica (TPC _F *. elastica*_). Factor coding is Coded. Sum of squares is Type III – Partial.

The F-value of 9.24 implied the model was significant. There was only a 0.01% chance that an F-value this large could occur due to noise. The P-values less than 0.0500 indicated that model terms were significant. In this case, X _2_, X _3_, X _1_X _4_, X _3_X _4_, X _4_², X _5_² were significant model terms. Values greater than 0.1000 indicate the model terms are not significant. If there were many insignificant model terms (not counting those required to support hierarchy), model reduction might be needed to improve the model. The lower lack of fit F-value of 0.95 implied the lack of fit was not significant relative to the pure error. There was a 57.65% chance that a lack of fit F-value this large could occur due to noise. Non-significant lack of fit demonstrates the model fits. The low lack of fit F-value and the high p-value indicated the reliability of the model that was generated.

***Fit statistics.*** The predicted R² of 0.5917 was in reasonable agreement with the adjusted R² of 0.7707; i.e., the difference was less than 0.2. The adequate precision measured the signal-to-noise ratio. A ratio greater than 4 was deemed desirable, which implied an adequate signal and that the model could be used to navigate the design. The higher the adequate precision the more suitable the model for optimization ^52^. In this study, the ratio of 12.620 indicated an adequate signal. This model could therefore be used to navigate the design space ( Table 5).

**Table 5.** Fit statistics.

**Std. Dev.**	14.43		**R²**	0.8643
**Mean**	67.11		**Adjusted R²**	0.7707
**C.V. %**	21.50		**Predicted R²**	0.5917
			**Adeq. precision**	12.6201

**Std. Dev.: standard deviation, C.V.%: percentage coefficient of variation, Adeq. precision: adequate precision; R ^2:^ coefficient of determination.**

The terms that were not statistically significant were excluded from the second-order polynomial to improve the model’s predictability. The coefficients of regression indicated that some of the linear terms had a positive effect on the response ( Table 6). The interaction terms between ethanol concentration and solid-liquid ratio, extraction time and temperature, extraction time and solid-liquid ratio, and pH and solid-liquid ratio had a positive impact on the response. The linear term, temperature, had the biggest effect on the response followed by extraction time and pH.

**Table 6.** Coefficients in terms of coded factors.

Factor	Coefficient estimate	df	Standard error
Intercept	88.05	1	5.06
X _1_-Ethanol concentration	-3.89	1	2.19
X _2_-Extraction time	8.78	1	2.19
X _3_-Temperature	18.26	1	2.19
X _4_-pH	1.86	1	2.19
X _5_-Solid-liquid ratio	-2.99	1	2.19
X _1_X _2_	-0.7135	1	2.55
X _1_X _3_	-0.0690	1	2.55
X _1_X _4_	-12.99	1	2.55
**X _1_X _5_**	2.40	1	2.55
**X _2_X _3_**	4.07	1	2.55
X _2_X _4_	-1.36	1	2.55
**X _2_X _5_**	1.83	1	2.55
X _3_X _4_	-5.38	1	2.55
X _3_X _5_	-0.8992	1	2.55
**X _4_X _5_**	1.02	1	2.55
X _1_²	-1.53	1	1.94
X _2_²	-0.9999	1	1.94
X _3_²	-1.92	1	1.94
X _4_²	-14.32	1	1.94
X _5_²	-5.41	1	1.94

**Figure 2.**
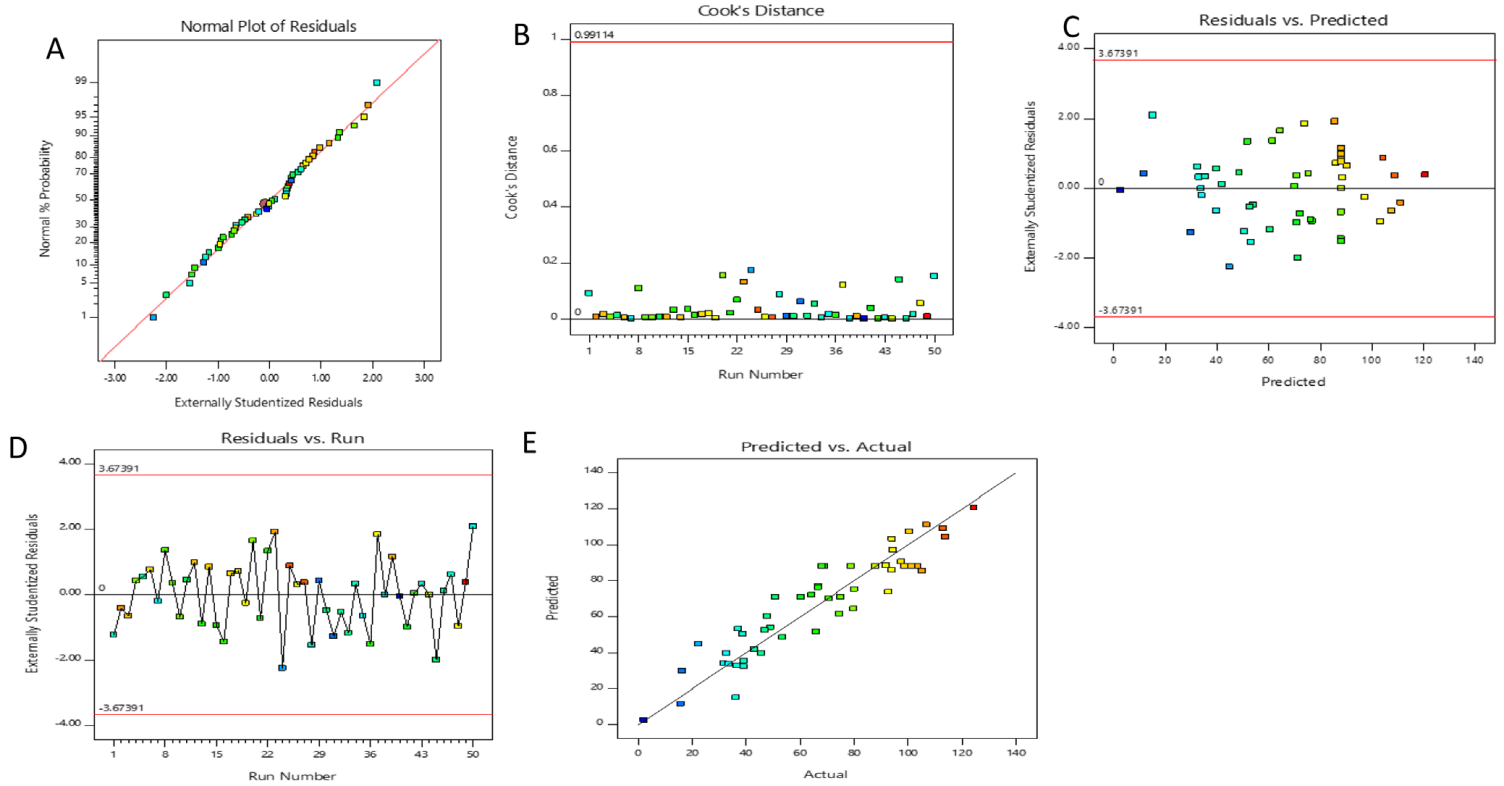
**a**) Normal probability plot of externally studentized residuals for the reduced polynomial model. **b**) Cook’s distance for the reduced polynomial model. **c**) The plot of residuals against predicted values. **d**) The plot of residuals against run number. **e**) Plot of predicted values against the observed values.

Diagnostic plots are illustrated in Figure 2a–e. The residuals were also used to determine the adequacy of the model, which characterizes the difference between the actual and predicted values of the response ^53^. The minor deviation of points from the straight line and even distribution of the residuals around it endorsed the normal distribution of the residuals, which validated the ANOVA in the reduced model and thereby indicated better prediction of the regression model. The normal plot of externally studentized residuals ( Figure 2a) shows the plot of the attained residuals versus expected values. The normally distributed residuals are suggestive of the obtained plots of the model after non-statistically significant terms are omitted.

The Cook’s distance values are shown in Figure 2b. There were no outliers in the given experimental dataset, which meant that Cook’s distances were less than the limit of 1.0. The plot of residuals against predicted values and against the run number is displayed in Figure 2c and d. The data points show unidentifiable patterns in these plots as they randomly fall on both sides of 0. These results specify the independence of the residuals from one another, their random distribution, and constant variance ^54^.

The plot of predicted versus actual values shown in Figure 2e presents data points that lie close to the straight line, indicating an adequate agreement between the actual data from the experiment and the predicted data from the mathematical model. Furthermore, the residuals for the response predictions are less than 5%. Therefore, a suitable model was suggested for characterizing the effects of extraction conditions for total phenolic recovery of *F. elastica* based on the results of the model adequacy diagnostic plots.

**Figure 3.**
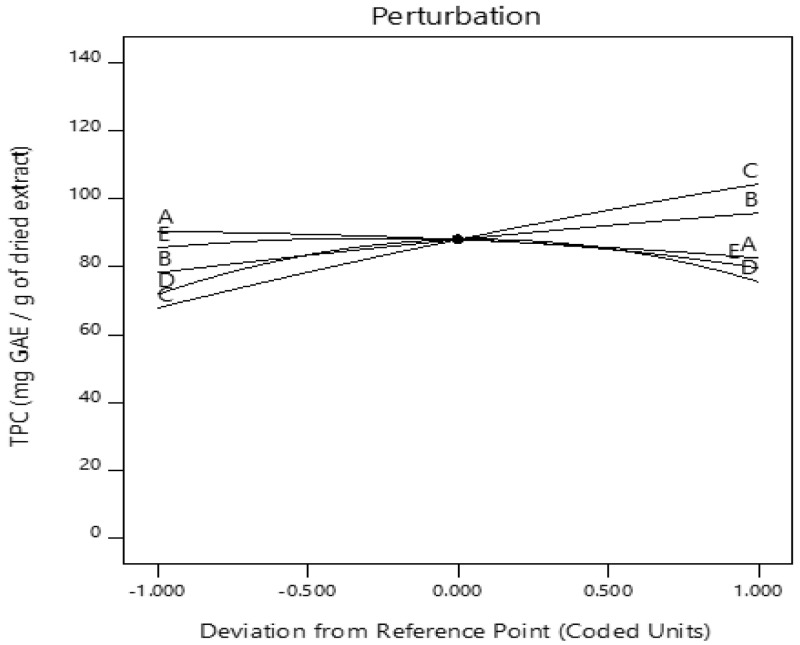
The perturbation plot displaying the impact of all the independent factors on the total phenolic content (TPC) at the center. A) ethanol concentration; B) time; C) temperature; D) pH; E) solid-liquid ratio.

***Optimization of the extraction conditions.*** The perturbation plot shows the effect of all the factors on a single plot. The perturbation plot ( Figure 3) indicates the impact of all the independent factors on the TPC at the center. The response is plotted by changing only one factor over its range while holding all the other factors constant. A variable with a steep slope or curvature indicates the sensitivity of the response to it, and a relatively flat line shows insensitivity to change in that particular factor ^55^.

It can be observed that TPC increased as time (B) and temperature (C) were increased, while an increase in ethanol concentration (A) resulted in a decrease in TPC content. The effect of a reduction in solid-liquid ratio on TPC is minimal. By evaluating the slope patterns, it was observed that pH and solid-liquid ratio did not have a linear impact compared to the other three factors. From the plot, pH has a strong effect on TPC recovery.

Exposure to severe acidic and alkaline conditions could result in oxidation and epimerization of the phenols.

**Figure 4.**
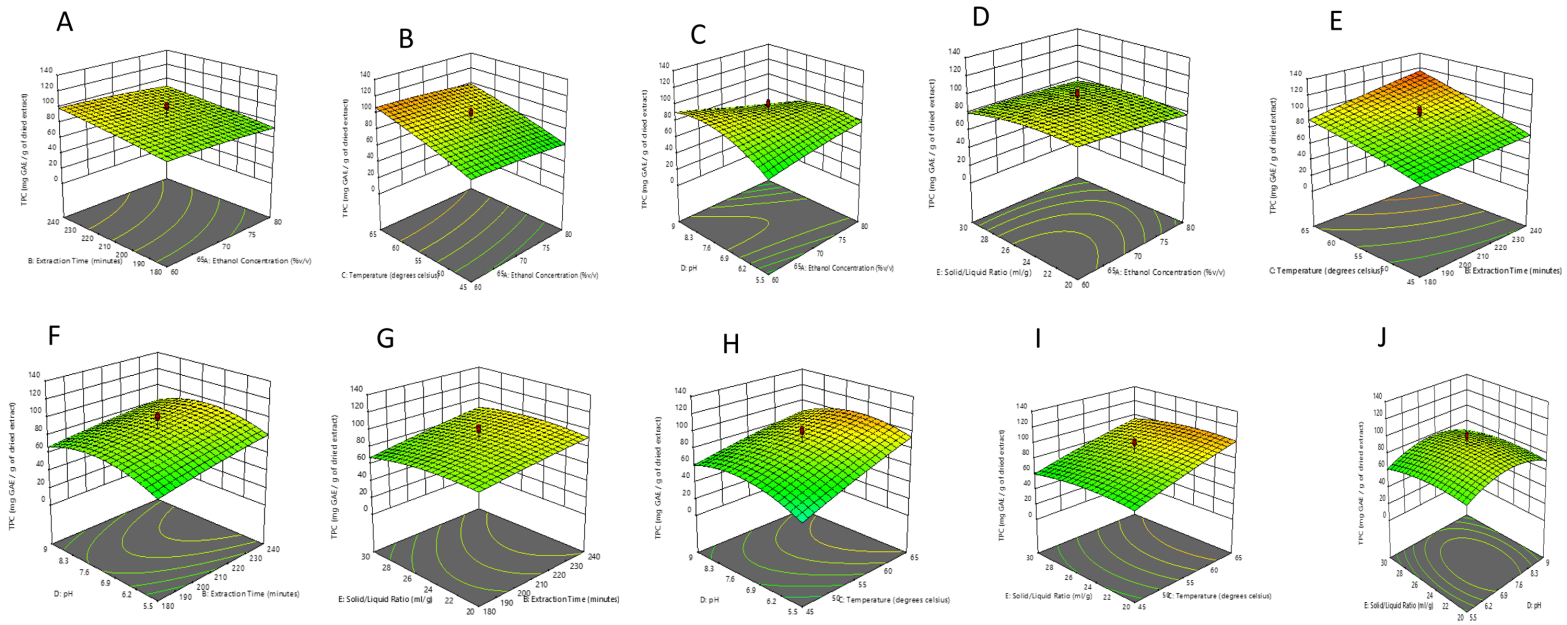
The interactive effect of **a**) of extraction time and ethanol concentration; **b**) extraction temperature and ethanol concentration; **c**) pH and ethanol concentration; **d**) solid-liquid ratio and ethanol concentration; **e**) temperature and extraction time; **f**) pH and extraction time; **g**) solid-liquid ratio and extraction time; **h**) pH and extraction temperature; **i**) solid-liquid ratio and extraction temperature; and **j**) solid-liquid ratio and pH.

***Analysis of the surface plots.*** The interaction of two of the five variables and their effect on TPC recovery while the rest of the three variables were kept constant is illustrated in the three-dimensional RSM plot. ( Figure 4a–j).

The interactive effect of extraction time and ethanol concentration whilst fixing pH, temperature, and the solid-liquid ratio at a constant level was investigated in Figure 4a. The TPC value increased slightly as the ethanol concentration ranged from 60-70% v/v and extraction time increased, with the maximum TPC content peaking at 98.85 mg GAE/g of dried extract at an ethanol concentration of 60% v/v and extraction time of 239.4 minutes. Khedher *et al.* (2017) used RSM to describe the effect of ethanol concentration and time of extraction on polyphenolic extraction of *C. asiatica* leaves. From their results, they observed a curved relationship between ethanol concentration and total phenolic extraction. Lower ethanol concentration resulted in a higher yield of total phenols. However, they reported extraction time as not having any significant impact on the response. Their results showed that at 30% ethanol concentration and duration of 90 minutes, the phenolic level peaked at 4.13 mg GAE/g dried weight of extract ^56^.

The interaction between temperature and ethanol concentration is depicted in Figure 4b. A similar linear effect is observed concerning temperature and caused an increase in the TPC. As usual, the TPC recovery increased with the decrease in the ethanol concentration. The optimum TPC content of 106.768 mg GAE/g of dried extract was observed at 60% v/v ethanol concentration and a temperature of 64.9°C. Using RSM analysis, Gajic *et al.* (2019) also reported a strong interaction between ethanol concentration and temperature at an extraction time of 25 minutes for the ultrasound-assisted extraction of phenolic compounds from black locust ( *Robiniae pseudoacaciae*) *.* A temperature increase utilizing lower ethanol concentrations led to higher TPC values ^57^.

In Figure 4c, the interactive effect between pH level and ethanol concentration is displayed in the 3D plots. A similar effect of ethanol concentration on the value of the response was observed. The TPC recovery increased as ethanol concentration ranged between 60-70% v/v. As for the pH, the TPC value gradually increased as the pH was rising. When the pH exceeded 8.2, the TPC value slightly decreased. Thus, the pH had a slight quadratic trend. The highest recovery of 94.182 mg GAE/g of dried extract was recorded at a pH of 8.16 and ethanol concentration of 60% v/v. Roselló-Soto *et al.* (2019) described the influence of pH and ethanol concentration on obtaining phenolics from the by-products of tiger nuts via conventional extraction methods using an ethanol: water mixture using RSM. The phenolic content was strongly affected by ethanol concentration (p = 0.0007). However, pH did not show a great influence on the phenolic extraction (p = 0.7961) ^58^.

The impact of solid-liquid ratio and ethanol concentration on the TPC is illustrated in the response surface 3D plot in Figure 4d, at a constant pH of 7.25, extraction time of 210 minutes, and temperature of 55°C. The TPC was less affected by the solid-liquid ratio and increased by decreasing the ethanol concentration. The maximum TPC of 91.7257 mg GAE/g of dried extract was observed at 60% ethanol concentration and 1:22.8 g/ml solid-liquid ratio. Gavrilović *et al.* (2018) reported the interaction between ethanol concentration and solid-liquid ratio as having a major impact on TPC recovery from the leaves of *Juglans nigra*. According to their results, TPC levels increased accordingly with an ethanol concentration ranging from 0 to 50% v/v, above which a decline in TPC levels was observed. The TPC yield also increased with an increase in solid-liquid ratio. Ethanol concentration and solid-liquid ratio were deemed to be one of the major parameters that affected ultrasound-assisted extraction ^59^.

Figure 4e illustrates the effect of the interaction between extraction temperature and extraction time on the TPC. Both variables showed a linear effect on the response. At a temperature of 55°C, and a time of 210 minutes, the total phenolic recovery was 88.0548 mg GAE/g of dried extract, and the optimal TPC content of 106.081 mg GAE/g of dried extract occurred at 65°C and 240 minutes. Zulkifli *et al.* (2020) reported the interaction between the time of extraction and temperature on TPC recovery of *Hylocereus polyrhizus* seed extract by setting the solvent concentration at 70% v/v. The team observed that TPC recovery efficacy was enhanced as temperature and time were concurrently increased ^60^. Kaleem *et al.* (2019) also investigated the effect of time and temperature on total polyphenolic extraction whilst keeping the solvent concentration fixed at 50% v/v. The TPC of the extract rose with increasing extraction time. However, after some time, the TPC level declined because phenolic compounds exposed to higher temperatures are destroyed over a longer period ^61^.

The interaction between extraction time and pH while keeping the other variables fixed is shown in Figure 4f. The increase in extraction time caused a similar influence in increasing the TPC value. The response gradually increased when the pH increased within a certain range. When the pH exceeded about 7.38, the TPC began to decrease slightly. At a pH of 7.38 and time of 239.8 minutes, the TPC was 95.7678 mg GAE/g of dried extract.

In a study by Skrypnik and Novikova (2020), the optimal yield of total phenolics was seen in the midpoint at a pH of 4 and the levels of total phenolic compounds analyzed in the extracts of apple pomace-based non-ionic emulsifiers was increased when time for extraction was increased from 40 minutes to 65 minutes, yet declined gradually when the time period was increased further ^20^.

The influence of extraction time and solid-liquid ratio on TPC is also illustrated in the response surface at constant ethanol concentration (70% v/v), extraction temperature (55°C), and pH of 7.25 ( Figure 4g). It revealed that the TPC was less affected by the solid-liquid ratio and increased with time when the other factors were kept constant at the center point. The maximum TPC content was found to be 96.6589 mg GAE/g of dried extract at an extraction time of 240 minutes and a solid-liquid ratio of 1:25 g/mL. The relationship between TPC and the interaction between the independent variables i.e., solid-liquid ratio and extraction time, was previously shown by Andres *et al.* (2020). It was found that the optimal TPC levels occurred within the range of 10-13 g/mL. Time of extraction didn’t have significant impact on the TPC ^62^.

The impact of temperature and pH on the response whilst keeping other factors fixed at the midpoint is shown in Figure 4h. The temperature has a positive linear effect on the TPC. The TPC gradually increased when the pH increased to a certain extent and when the pH exceeded about 7.20, there was a slight decrement in the TPC value. The optimum TPC recovery of 104.441 mg GAE/g of dried extract was at a temperature of 65°C and pH of 7.20.

An interactive effect between pH and temperature was reported by Roselló-Soto *et al.* (2019) using RSM. They reported an optimum TPC level at a pH of 7 and a temperature of 37°C at a constant ethanol concentration ^58^.

The three-dimensional response surface plot illustration of the interaction between temperature and solid-liquid ratio is shown in Figure 4i. The TPC increased at higher extraction temperatures and was less affected by the change in solid-liquid ratio at constant pH (7.25), time (210 minutes), and ethanol concentration (70% v/v). At a temperature of 57.5°C and solid-liquid ratio of 1:23 g/mL , the TPC was 92.93 mg GAE/g of dried extract, and the optimal TPC of 104.958 mg GAE/g of dried extract occurred at 65°C and a 1:22.6 solid-liquid ratio. The impact of the interactions between solid-liquid ratio and temperature has been investigated by Radojkovic *et al.* (2013) for optimization of total phenolic yield from mulberry extracts employing RSM. From their 3D plots, the TPC increased with increasing temperature to about 65°C, after which subsequent temperature increase didn’t cause any significant change in TPC. The author also observed a significant increase in TPC as solid-liquid ratio was increased, reaching a maximum at about 1: 20 g/mL. Further increase in the solid-liquid ratio did not yield any significant impacts on TPC levels, however. The TPC levels of mulberry extracts varied from 18.63 to 52.43 mg of GAE/g of dried extract ^63^.

The responses observed for the effect of the interaction between pH and the solid-liquid ratio at a fixed ethanol concentration of 70% v/v, temperature of 55°C, and time of 210 minutes indicated that solid-liquid ratio has less effect on ensuring maximum TPC ( Figure 4j). For example, at a pH of 7.36 and solid-liquid ratio of 1: 23.18, the TPC was 88.463 mg GAE/g of dried extract.

In the optimization of anthocyanin extraction, 3D plots displayed a steeper change in pH in comparison to the solid-liquid ratio, signifying pH has a greater impact, which is consistent with our results ^64^.

### Determination of optimal conditions and validation of model

For the verification of the model’s accuracy for optimal yield prediction, actual experiments were performed using the optimal extraction conditions. The values were very close to the predicted values, which indicated the reliability of the optimization accomplished in the research. The quadratic polynomial regression model that was generated gave the optimal extraction conditions with a desirability of 1.000. Desirability functional values lie between 0 and 1. The value 0 is ascribed when the variables give an undesirable response, whereas the value 1 parallels the optimal functioning for the studied variable ^65, 66^. The optimal conditions for extracting total phenols from *F. elastica* hydroethanolic extracts were as follows; ethanol concentration 75.98% v/v, extraction time of 193.9 minutes, temperature of 63.66°C, pH of 5.63, and solid-liquid ratio of 1:25.12. At these optimal conditions, the TPC was 89.467 mg GAE/g of dried extract ( Table 7).

**Table 7.** Comparison of the predictive and the experimental result optimum values of TPC recovery.

Response	Optimal conditions ^1^					Maximum yield	
	X _1_	X _2_	X _3_	X _4_	X _5_	Predicted	Actual
TPC	75.99	193.86	63.66	5.62	1:21.12	89.467 mg GAE/g of dried extract	82.83 ± 3.335 mg GAE/g of dried extract

^1^X _1_: ethanol concentration (%); X _2_: Time; X _3_: temperature (°C); X _4_: pH. X _5_: solid-liquid ratio. TPC, total phenolic content; GAE, gallic acid equivalent. Experimental results were expressed as mean values ± standard deviation (n=6).

To verify the model’s capability to accurately predict the actual value, six replicates of verification experiments were undertaken and the outcome was 82.83 ± 3.335 mg GAE/g of dried extract, which was very close to the predicted value.

## Conclusions

The extraction of polyphenols from the most active extract, the ethanolic extract of *F. elastica* was undertaken in this study. The extraction was performed via maceration, and the relationship between the total phenolic recovery and experimental variables, which included ethanol concentration, extraction time, extraction temperature, solid-liquid ratio, and pH, was considered. A multiple single-factor analysis was performed. All the extraction factors exhibited a significant (p <0.05) effect on TPC recovery of the *F. elastica* ethanolic extract. Optimization with the RSM procedure was performed concerning the five selected parameters. The optimal conditions that gave maximum TPC are as follows; ethanol concentration of 75.99% (v/v), extraction time of 193.86 minutes, extraction temperature of 63.66°C, pH of 5.60, and solid-liquid ratio of 1:21.12 g/mL. The model validation revealed that these conditions gave a TPC recovery of 82.83 ± 3.335 mg GAE/g of dried extract. Future studies should focus on scalability and feasibility assessments of maceration production of biologically active compounds by employing new optimization techniques, such as artificial neural networks, etc.

## Data availability

### Underlying data

Harvard Dataverse: Replication Data for Optimization of Extraction Conditions for Polyphenols from the stem bark of *Funtumia elastica* (Funtum) using Response Surface Methodology https://doi.org/10.7910/DVN/K0WZZL ^28^.

This project contains the following underlying data:

- Analysis FIT summary.tab
- ANOVA.tab
- Experimental Design Conditions.tab
- Single Factor Analysis.tab
- Data showing concentrations and absorbances for Actual Optimal Runs.tab
- Data showing concentrations and absorbances for Experimental Runs.tab
- Data showing concentrations and absorbance for Single Factor Analysis.tab

Data are available under the terms of the Creative Commons Zero "No rights reserved" data waiver (CC0 1.0 Public domain dedication).
