# Supplementary material for: Optimization of extraction conditions for polyphenols from the stem bark of Funtumia elastica (Funtum) utilizing response surface methodology
Source: AAS Open Res. 2021 Oct 25;4:46. Originally published 2021 Sep 10. [Version 2] doi: 10.12688/aasopenres.13284.2 (PMC8479850; doi:10.12688/aasopenres.13284.2)
Supplement: Peer review history

# Peer review: Reviewer response for version 2

**DOI:** 10.21956/aasopenres.14446.r28972

**Published:** 2021-11-02

**Authors:** Adewale Adewuyi

**Affiliations:** 1 Department of Chemical Sciences, Redeemer's University, Ede, Nigeria

Thank you for the opportunity. Kindly accept the paper in its current form. Approved.

Is the work clearly and accurately presented and does it cite the current literature?

Yes

If applicable, is the statistical analysis and its interpretation appropriate?

Yes

Are all the source data underlying the results available to ensure full reproducibility?

Yes

Is the study design appropriate and is the work technically sound?

Yes

Are the conclusions drawn adequately supported by the results?

Yes

Are sufficient details of methods and analysis provided to allow replication by others?

Partly

Reviewer Expertise:

NA

I confirm that I have read this submission and believe that I have an appropriate level of expertise to confirm that it is of an acceptable scientific standard.

# Peer review: Reviewer response for version 1

**DOI:** 10.21956/aasopenres.14408.r28876

**Published:** 2021-09-29

**Authors:** Adewale Adewuyi

**Affiliations:** 1 Department of Chemical Sciences, Redeemer's University, Ede, Nigeria

The authors have presented an interesting work which is a contribution to scientific knowledge. Please see my comments below:

**Abstract:**

*Method:*

- “The effect of the extraction factors on the phenolic content was statistically significant (p <0.05)”. This sentence sounds more like a result. The authors should either delete or reword to reflect the intended statistical tool used for analysis.

*Conclusion:*

- The conclusion did not address the objective which is “optimization of factors that affect the efficiency for the extraction of phenolic compounds from the stembark of *Funtumia elastic”. *The aim is the optimization of extraction process. Therefore, the authors should indicate in the conclusion whether the proposed optimization improved extraction process for extraction of phenolic compounds *.*

**Introduction:**

 Using few words, the authors should kindly indicate in the introduction the reasons for using central-composite-design (CCD) of response-surface-method. They should include a clear justification or novelty that comes with CCD; e.g advantages of using CCD that makes is suitable in the current study.

*Methods:*

- Change 'botanist' to 'Botanist'.
- Write 'KNUST' in full.

*Reagent:*

- Change 'obtained' to 'purchased'.

*Maceration extraction:*

- The authors should be specific with the volume of Erlenmeyer conical ﬂasks used. 250 or 500 mL? If this was in different volumes what informed this?
- The authors should be more detailed with the method used since there was no reference to any previous method. The authors should indicate whether the mixture was filtered, how was the extracting solvent (hydroethanolic solution) removed, did you use a rotary evaporator or how? How long was the extraction for? In the case of time just indicate the maximum period under maceration extraction because this kinetic was mentioned in Single-factor analysis. The authors should indicate whether absolute ethanol was used in preparing the hydroethanolic solution.

*Determination of TPC:*

- Change '10 mins' to '10 min'.

**Experimental design:**

- Change 'group into two' to 'grouped into two'.

**Single-factor analysis:**

- Change “time for extraction (60, 120, 180, 240, and 300 minutes)” to time for extraction (60, 120, 180, 240, and 300 min). The authors should be consistent with the use of unit.

***RSM:***

- Change time (138.65 - 281.35 minutes) to time (138.65 - 281.35 min).

**Results and discussion:**

 The authors should check and strictly follow the referencing style. The authors used both author centered and numbering e.g.:

*Effect of the ethanol concentration.*

1. "Chew *et al. *(2011) performed...".

*Effect of extraction time*

1. "Jia *et al. *(2019) reports the...".
2. "Le *et al. *(2019) also...".

*Effect of solid-liquid ratio*

1. "Wang *et al. *(2020) also demonstrated...".

The authors should carefully read through the manuscript and correct the referencing style by abiding with the journal style of referencing.

**Tables and Figures:**

- The author should improve on the quality of the tables and figures presented.

Is the work clearly and accurately presented and does it cite the current literature?

Yes

If applicable, is the statistical analysis and its interpretation appropriate?

Yes

Are all the source data underlying the results available to ensure full reproducibility?

Yes

Is the study design appropriate and is the work technically sound?

Yes

Are the conclusions drawn adequately supported by the results?

Yes

Are sufficient details of methods and analysis provided to allow replication by others?

Partly

Reviewer Expertise:

Chemistry

I confirm that I have read this submission and believe that I have an appropriate level of expertise to confirm that it is of an acceptable scientific standard, however I have significant reservations, as outlined above.

# Peer review: Reviewer response for version 1

**DOI:** 10.21956/aasopenres.14408.r28874

**Published:** 2021-09-28

**Authors:** Steven Bloom

**Affiliations:** 1 Department of Medicinal Chemistry, University of Kansas, Lawrence, KS, USA

Phenolic compounds act as natural antioxidants and are directly involved in lipid peroxidation and anti-carcinogenesis. Phenolic compounds also exhibit anti-inflammatory, antimicrobial, cardio-protective, and antiaging activities. In order to better study the biological (potential therapeutic) activity of phenolic compounds, this requires reliable methods to isolate them on-scale, one possibility being from natural sources. The stembark of *Funtumia elastica* is a rich source of phenolic compounds, and a prime candidate on which to develop an efficient extraction protocol.

 To optimize the extraction of phenolic compounds from *Funtumia elastica*, the effect of five independent variables (i.e., EtOH solvent concentration, time, temperature, solid-liquid ratio, and pH) on the amount of phenolic compound extracted were examined separately (holding the other independent variables constant). The Folin-Ciocalteu reagent was used to determine the total phenolic content extracted. Optimal ranges found for each independent variable were then combined using the central-composite-design of the response surface method (RSM) to determine the best overall extraction conditions (i.e., conditions where each independent variable is optimal). For the verification of the model’s accuracy actual experiments were performed using the predicted optimal extraction conditions. The actual values of extracted phenolic compounds were very close to the predicted values, which indicates the reliability of the optimization accomplished in the research. Thus, the conditions reported herein are very useful for extracting phenolic compounds from *Funtumia elastica *and this study provides a blueprint for designing extraction protocols from other natural sources in the future (for phenolic compounds or otherwise).

 Overall, the results are well organized, and the significance of the results are well supported by statistical analysis (F-test, P-values, ANOVA, etc.). The statistical analysis of each independent variable and of the overall extraction conditions adds considerable confidence to the optimization procedure and its results. The authors also provide a thorough analysis of their results, comparing them to previous reports from other groups. This level of critical analysis adds more confidence to the work and in the results obtained from the study.

Is the work clearly and accurately presented and does it cite the current literature?

Yes

If applicable, is the statistical analysis and its interpretation appropriate?

Yes

Are all the source data underlying the results available to ensure full reproducibility?

Yes

Is the study design appropriate and is the work technically sound?

Yes

Are the conclusions drawn adequately supported by the results?

Yes

Are sufficient details of methods and analysis provided to allow replication by others?

Yes

Reviewer Expertise:

Synthetic organic chemistry, peptide chemistry, photochemistry, and HIV-virology

I confirm that I have read this submission and believe that I have an appropriate level of expertise to confirm that it is of an acceptable scientific standard.

# Response

**Published:** 2021-09-30

**Authors:** Mercy Badu

**Affiliations:** Kwame Nkrumah University of Science and Technology, Ghana

No comment.

# Peer review: Reviewer response for version 1

**DOI:** 10.21956/aasopenres.14408.r28873

**Published:** 2021-09-24

**Authors:** Samson A. Abagale, Ohene Boansi Apea

**Affiliations:** 1 Department of Applied Chemistry and Biochemistry, Faculty of Applied Sciences, CK Tedam University of Technology and Applied Sciences, Navrongo, Ghana; 2 CK Tedam University of Technology and Applied Sciences, Navrongo, Ghana

**Abstract:**

- The effect of the extraction factors on the phenolic content was statistically significant (p <0.05).

Presented under 'Methods' but is an outcome (Results). *It is recommended that* this should rather be stated as saying that the statistical significance of the “effect of the extraction factors on phenolic content” was tested and the outcome enabled selection of the extract for further work.

- "For the response surface method, a five/factor, five/level central composite design **was** used..."

*It is recommended that* “was” be inserted.

- Conclusion - The conclusion falls short of embracing the topic’s interest of ‘Optimization of extraction conditions”. The conclusion statement is good but the current conclusion needs to be augmented in line with the topic and findings in the study. 

Also, the effect of the parameters stated as a "purpose of the study" in the last paragraph of the introduction is entirely left out in this summary of conclusion. *It is recommended that *the conclusion should embody the essence of the study.

**Introduction:**

- “It is therefore imperative to assess the interaction between the independent variables” at the beginning of paragraph 6;

*It is recommended* that this should actually be at the end of paragraph 4 as it is a justification of the project based on what was stated as challenges in paragraph 4.

 “The purpose of the study was to evaluate the effects of the investigated factors…”

**Third paragraph under Results:**

 "When solvents of different polarities are mixed, they tend to extract a wide spectrum of compounds ^30^." *It is recommended that* “wide” be substituted with “wider”.

**Effect of extraction pH:**

1. can modify the phenolic compounds’ chemical structure and thus the antioxidant activity of the compounds.

*It is recommended that* this needs revision to carry the meaning purported.

 2. The antioxidant properties are pH-dependent, as changes in pKa values are consistent with changes in hydroxyl ionization

 or other functional groups of the phenolic compounds ^20^.

 This is hanging. The sentence is not enough to sit as a paragraph, yet it is unrelated to the paragraph before it. *It is recommended that *it should be revised or otherwise removed entirely.

**Conclusion:**

 "The extraction of polyphenols from the most active extract, the ethanolic extract of *F. elastica *was undertaken in this study. The extraction was performed via maceration, and the relationship between the total phenolic recovery and experimental variables, which included ethanol concentration, extraction time, extraction temperature, solid-liquid ratio, and pH, was considered. A multiple single-factor analysis was performed." "...Optimization with the RSM procedure was performed concerning the five selected parameters. The optimal conditions ... as follows; ... Future studies should focus on ... networks, etc."

 The conclusion is presented as if it is an abstract. Take note of the highlighted portions above. They are indicative of activity already presented previously in earlier part of the paper. Thus, experimental approaches should be removed and their final outcomes/findings rather reported. For example: Using maceration, the ethanol extract of *F. elastica *was identified as the most active extract. From the studies between the total phenolic recovery and experimental variables studied, all the extraction factors exhibited a significant (p <0.05) effect on TPC recovery of the *F. elastica *ethanolic extract. Optimisation with the RSM procedure concerning the five selected parameters gave maximum TPC are as follows; ….

1. The modeling forms a significant aspect of the work. But no reference conclusion is made on it. At least its success should be stated.
2. “etc” ending should be removed. If other factors could be stated in addition it will be good, otherwise it is fine as stated without the “etc”.

Is the work clearly and accurately presented and does it cite the current literature?

Partly

If applicable, is the statistical analysis and its interpretation appropriate?

Yes

Are all the source data underlying the results available to ensure full reproducibility?

Yes

Is the study design appropriate and is the work technically sound?

Yes

Are the conclusions drawn adequately supported by the results?

Partly

Are sufficient details of methods and analysis provided to allow replication by others?

Yes

Reviewer Expertise:

Applications of principles of chemistry, involving chemical ecology and related areas, for the protection and well being of humans, animals, crops and the environment.

We confirm that we have read this submission and believe that we have an appropriate level of expertise to confirm that it is of an acceptable scientific standard.
